# CPX-351 Pharmacokinetics and Safety in Adults with Hematologic Malignancies and Renal Function Impairment: Phase 1 Trial

**DOI:** 10.3390/cancers16050915

**Published:** 2024-02-24

**Authors:** Scott R. Solomon, Bayard L. Powell, Jamie Koprivnikar, Catherine Lai, Heather Male, Laura C. Michaelis, Laura F. Newell, David Sanford, Jack Jenkins, Amy Zelaya, Sheryl Coppola, Stefan Faderl, Roland B. Walter

**Affiliations:** 1 Bone & Marrow Transplant (BMT), Leukemia and Cellular Immunotherapy Programs, Northside Hospital Cancer Institute, Atlanta, GA 30342, USA; 2 Atrium Health Wake Forest Baptist Comprehensive Cancer Center, Winston-Salem, NC 27157, USA; 3 John Theurer Cancer Center at Hackensack Meridian Health, Hackensack, NJ 07601, USA; 4 Abramson Cancer Center, University of Pennsylvania, Philadelphia, PA 19104, USA; 5 University of Kansas Cancer Center, Kansas City, KS 66160, USA; 6 Division of Hematology/Oncology, Froedtert Hospital, Medical College of Wisconsin, Milwaukee, WI 53226, USA; 7 Knight Cancer Institute, Hematology and Medical Oncology, Oregon Health & Science University, Portland, OR 97239, USA; 8 Leukemia and Bone Marrow Transplant Program of British Columbia, Division of Hematology, Department of Medicine, University of British Columbia, Vancouver, BC V5Z 1M9, Canada; 9 Jazz Pharmaceuticals, Philadelphia, PA 19103, USA; 10 Jazz Pharmaceuticals, Palo Alto, CA 94304, USA; 11 Translational Science and Therapeutics Division, Fred Hutchinson Cancer Center, Seattle, WA 98109, USA

**Keywords:** CPX-351, hematologic cancer, pharmacokinetics, renal impairment

## Abstract

**Simple Summary:**

CPX-351 has been approved for the treatment of acute myeloid leukemia. Previous studies have indicated that dose adjustment is not warranted for patients with mild to moderate renal impairment. However, the effect of severe renal impairment on the use of CPX-351 has not been established. In this study, we evaluated CPX-351 in patients with normal renal function and in patients with moderate and severe renal impairment. We compared how CPX-351 moves through the body, as well as the safety of the drug, in these different patient groups. The results suggest that CPX-351 dose adjustment is not required for patients with hematologic malignancies with moderate or severe renal impairment.

**Abstract:**

This open-label phase 1 study (clinicaltrials.gov, NCT03555955) assessed CPX-351 pharmacokinetics (PK) and safety in patients with hematologic malignancies with normal or impaired renal function. Patients were enrolled into three cohorts based on their creatinine clearance (CrCl): ≥90 mL/min (Cohort 1, normal renal function, *n* = 7), 30 to <59 mL/min (Cohort 2, moderate renal impairment, *n* = 8), or <30 mL/min (Cohort 3, severe renal impairment, *n* = 6). Patients received intravenous CPX-351 for initial induction; blood and urine samples were collected for PK analysis. The primary objective was to assess the PK parameters for cytarabine, daunorubicin, and their respective metabolites, arabinosyluracil (Ara-U) and daunorubicinol. Renal impairment did not significantly impact the cytarabine, daunorubicin, or daunorubicinol exposure, but it caused a slight increase in the Ara-U exposure. The CPX-351 side effect profile was similar in patients with impaired renal function compared to those with normal renal function. All the patients reported ≥1 treatment-emergent adverse event (TEAE), most commonly febrile neutropenia and nausea (57% each) and hyperglycemia (43%); no patients discontinued treatment due to TEAEs. These data suggest that CPX-351 dose adjustment is not required for patients with hematologic malignancies with moderate or severe renal impairment.

## 1. Introduction

Many patients with acute myeloid leukemia (AML), acute lymphoblastic leukemia (ALL), and high-risk myelodysplastic syndrome (MDS) have a poor long-term prognosis [1,2,3,4] and require therapies that maximize anti-tumor efficacy [3,5]. While conventional cytotoxic chemotherapeutics are generally thought to have maximal efficacy at the highest tolerable doses [3,6], many patients present with significant comorbidities, including renal impairment, which may require administering therapies at attenuated doses or even a transition to palliation-oriented therapy [7,8,9,10,11]. However, in such situations, data on how to renally adjust drug dosing are often missing or sparse [12]. This is partly because clinical trials commonly exclude patients with renal impairment, potentially preventing these patients’ access to drugs that might benefit them [13].

CPX-351 (United States/Canada: Vyxeos^®^; European Union/United Kingdom: Vyxeos^®^ liposomal) is a fixed-dose combination of daunorubicin and cytarabine maintained at a 1:5 molar ratio within a nanoscale liposomal delivery vehicle [7,14,15,16]. In the United States, CPX-351 is approved for newly diagnosed, therapy-related AML (t-AML) or AML with myelodysplasia-related changes (AML-MRC) in adult and pediatric (aged ≥1 year) patients [16]. In Canada, the European Union, and the United Kingdom, CPX-351 is approved for the treatment of newly diagnosed adults with t-AML or AML-MRC [15,17].

Previously, the results of a population pharmacokinetic (PK) analysis indicated that no dose adjustments of CPX-351 were needed for patients with hematologic malignancies and mild/moderate renal impairment [18]. However, the effect of severe renal impairment on the PK and side effect profile of CPX-351 has not been established. To address this unmet need, this phase 1 study evaluated the potential impact of moderate and severe renal impairment on the PK and safety of CPX-351 in patients with hematologic malignancies considered suitable for intensive chemotherapy.

## 2. Materials and Methods

### 2.1. Study Design and Patients

This phase 1 open-label study (NCT03555955) was conducted at 10 centers across North America. Eligible patients were ≥18 years old. In order to address the unmet need for salvage therapy options for patients with hematologic malignancies, the eligibility criteria were deliberately broad to allow for the inclusion of patients with newly diagnosed, primary refractory, or relapsed AML, ALL, and MDS, and to improve the feasibility of the study. Other requirements included an Eastern Cooperative Oncology Group (ECOG) performance status of 0–2 and suitability for intensive chemotherapy. This analysis excluded patients with newly diagnosed acute promyelocytic leukemia (APL) t(15;17) or core-binding factor (CBF) leukemia, including t(8;21) or inv(16), if known at the time of registration; patients with APL or CBF leukemia in first or subsequent relapse were eligible for enrollment. Also excluded were patients who had prior cumulative anthracycline exposure of >500 mg/m^2^ daunorubicin (or equivalent) and patients who were undergoing dialysis.

Patients were assigned to one of three cohorts based on their level of creatinine clearance using the Cockcroft–Gault formula [19]. Cohort 1 included patients with creatinine clearance of ≥90 mL/min (normal renal function), Cohort 2 included patients with creatinine clearance of 30 to <59 mL/min (moderate renal impairment), whereas Cohort 3 included patients with creatinine clearance of <30 mL/min (severe renal impairment).

This study was conducted in accordance with the ethical guidelines of the Declaration of Helsinki, applicable International Council for Harmonization Good Clinical Practice Guidelines, and other applicable laws and regulations. Written informed consent was obtained from all the patients prior to enrollment.

### 2.2. Treatment

During induction, the standard dose of CPX-351 (daunorubicin 44 mg/m^2^ and cytarabine 100 mg/m^2^) was administered via 90 min intravenous (IV) infusion on days 1, 3, and 5. As the maximum tolerated dose (MTD) of CPX-351 in patients with severe renal impairment has not yet been established, expedited PK and safety results from the first three patients in Cohort 3 (severe impairment) at the full dose were used to help determine the dose of CPX-351 for the remaining patients in the cohort. A second induction treatment (full dose CPX-351: daunorubicin 44 mg/m^2^ and cytarabine 100 mg/m^2^ on days 1 and 3, or reduced dose CPX-351: daunorubicin 28.6 mg/m^2^ and cytarabine 65 mg/m^2^ on days 1 and 3) and consolidation treatment (full dose CPX-351: daunorubicin 29 mg/m^2^ and cytarabine 65 mg/m^2^ on days 1 and 3, or reduced dose CPX-351: daunorubicin 19.4 mg/m^2^ and cytarabine 44 mg/m^2^ on days 1 and 3) were permitted at the investigator’s discretion. The duration of the CPX-351 treatment varied from approximately 21 days to 224 days, depending on the number of courses administered (1 to 4).

### 2.3. Endpoints

The primary endpoint was to characterize the impact of moderate and severe renal impairment on the PK of CPX-351. Plasma PK samples were collected on day 1 and day 5 at predose (time 0). Subsequent PK samples were collected 45 min, 90 min, and 2, 3, 4, 6, 8, 24, 48, 96, 168, and 216 h after day 5 time 0. Urine samples were collected at the following three intervals after day 5 dosing: 0 to 8 h, 8 to 24 h, and 24 to 48 h. The following parameters were assessed in patients with normal renal function or moderate or severe renal impairment: area under the plasma concentration–time curve from time 0 to next dosing over 48 h on day 5 (AUC_tau_), maximum plasma concentration (C_max_), time to maximum plasma concentration (T_max_), terminal elimination half-life (T_½_), total clearance at steady-state (CL_ss_; calculated as dose/AUC_tau_), renal clearance (CLR), cumulative amount of drug or metabolite eliminated in the urine over 48 h (Ae), volume of distribution in terminal state (V_z_), and volume of distribution in steady state (V_ss_) for total daunorubicin and cytarabine.

The secondary endpoints included the PK parameters for the metabolites of daunorubicin and cytarabine (daunorubicinol [active metabolite] and arabinosyluracil [Ara-U, inactive metabolite], respectively) in patients with normal renal function or moderate or severe renal impairment: AUC_0–48h_ (area under the plasma concentration–time curve for metabolites from time 0 to 48-h post-day 5 dosing), C_max_, T_max_, T_½_, and Ae.

The secondary safety and tolerability endpoints included the incidence and severity of treatment-emergent adverse events (TEAEs) in all the patients. The study also assessed vital signs, electrocardiograms (ECGs), echocardiography/multiple gated acquisition scans (MUGAs), and clinical laboratory tests (chemistry, hematology, coagulation, and urinalysis) in all the patients.

The efficacy was reported for the investigator-assessed post-induction unconfirmed responses.

### 2.4. Statistical Analysis

This study planned to enroll 18–24 patients to achieve 6–8 patients per cohort; there was no hypothesis testing and no control of error rates. The safety analysis set included all the enrolled patients who received ≥1 infusion of CPX-351. The PK analysis set included all the patients in the safety analysis set with ≥1 evaluable PK concentration, and the PK evaluable analysis set included all the patients in the PK analysis set whose key PK parameters could be determined.

The impact of renal impairment was evaluated using analysis of variance (ANOVA) for the natural logarithm (ln) transformed PK parameters (including AUC_tau_, CL_ss_, CLR, and C_max_) for daunorubicin and cytarabine only, with a term for the cohort as a fixed effect. Data were reported as geometric means, geometric mean ratios and 90% confidence intervals (CI) for the geometric mean ratios, and continuous PK data were summarized descriptively by analyte, course of treatment, date/time and by cohort, where appropriate. Safety and efficacy data were summarized descriptively, and categorical variables were summarized as counts and percentages.

## 3. Results

### 3.1. Patients

Overall, 21 adult patients were enrolled in this study between November 2018 and May 2021 from 10 centers (Supplementary Figure S1), with 18 patients completing the study. Of the three patients who discontinued the study, two were due to adverse events (one leading to death, deemed unrelated to the study drug); the reason for the third discontinuation is unknown. Most patients had newly diagnosed AML, were male, white, and of non-Hispanic or non-Latino descent; age was well balanced across the cohorts (Table 1).

### 3.2. Exposure

All 21 patients received a first induction course of CPX-351. While subsequent treatment cycles were permitted based on investigator discretion, no patients were determined to be candidates for a second induction course. Two patients received consolidation treatment. During the first induction course, the cumulative actual dose of the individual components of CPX-351 was similar for all the patients. The mean cumulative actual dose of daunorubicin for patients with normal renal function, moderate renal impairment, and severe renal impairment was 298.37 mg, 259.58 mg, and 240.75 mg, respectively, whereas the mean cumulative actual cytarabine dose for the same cohorts was 573.29 mg, 589.50 mg, and 548.00 mg.

### 3.3. Pharmacokinetics of CPX-351

Following treatment with CPX-351, cytarabine is metabolized by cytidine deaminase into the inactive metabolite Ara-U and daunorubicin is metabolized by carbonyl reductase into the active metabolite daunorubicinol.

#### 3.3.1. Cytarabine and Ara-U

**Figure 1:**
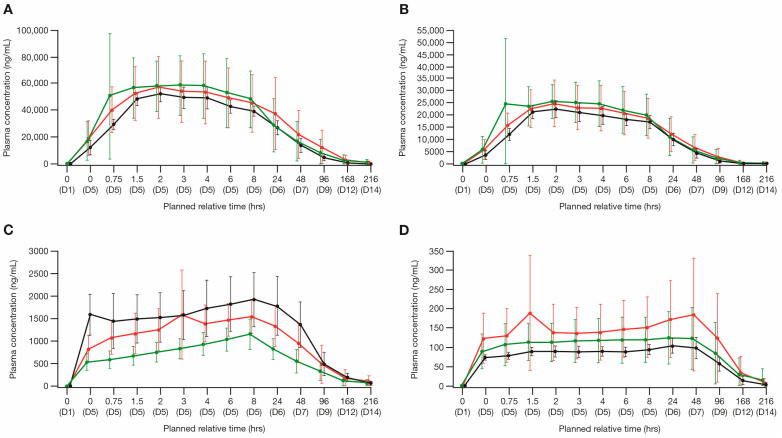
Arithmetic mean plasma PK concentration (±SD) of the individual components of CPX-351 and their metabolites (PK analysis set). Linear time plots by cohort of: (**A**) cytarabine, (**B**) daunorubicin, (**C**) Ara-U, and (**D**) daunorubicinol. Ara-U, arabinosyluracil; D, day; Hrs, hours; PK, pharmacokinetic; SD, standard deviation.

After the start of infusion on day 5, the plasma concentrations of cytarabine peaked at approximately 2–4 h, whereas the plasma concentrations of Ara-U peaked at approximately 8 h (Figure 1A,C). For cytarabine, the geometric mean AUC_tau_ values were 1,325,000 h × ng/mL for Cohort 1, 1,213,000 h × ng/mL for Cohort 2, and 1,330,000 h × ng/mL for Cohort 3. The geometric mean C_max_ values were 65,740 ng/mL for Cohort 1, 52,330 ng/mL for Cohort 2, and 52,120 ng/mL for Cohort 3. A summary of the plasma PK parameters for cytarabine and Ara-U is presented in Supplementary Table S1. The point estimate of the geometric mean ratio for cytarabine AUC_tau_ was approximately 0.38% higher for patients with severe renal impairment (Cohort 3) and 8% lower for patients with moderate renal impairment (Cohort 2) relative to patients with normal renal function (Cohort 1). The point estimate of the geometric mean ratio for cytarabine C_max_ was approximately 21% and 20% lower for patients with severe and moderate renal impairment (Cohort 3 and Cohort 2, respectively) compared with patients with normal renal function (Cohort 1). Based on the primary ANOVA analyses, the differences in exposure were within the observed variability of cytarabine AUC_tau_ and C_max_, suggesting that renal impairment did not have a clinically significant impact on the cytarabine exposure (Table 2). Slight increases in the Ara-U exposure (AUC_tau_ and C_max_) were observed with increasing levels of renal impairment (Supplementary Table S1). Both cytarabine Ae and CLR were low across all the cohorts, indicating the minimal contribution of renal elimination. The Ara-U Ae, cytarabine and Ara-U Ae_total, and cytarabine and Ara-U Ae%, were similar across all the cohorts (Table 3).

#### 3.3.2. Daunorubicin and Daunorubicinol

The plasma concentrations of daunorubicin peaked approximately 2 h after the start of infusion on day 5, whereas the plasma concentrations of daunorubicinol peaked around 48 h after the start of infusion on day 5 (Figure 1B,D). For daunorubicin, the geometric mean AUC_tau_ values were 524,100 h × ng/mL for normal renal function (Cohort 1), 495,000 h × ng/mL for moderate renal impairment (Cohort 2), and 508,600 h × ng/mL for severe renal impairment (Cohort 3). The geometric mean C_max_ values were 29,900 ng/mL for normal renal function (Cohort 1), 23,180 ng/mL for moderate renal impairment (Cohort 2), and 22,290 ng/mL for severe renal impairment (Cohort 3). A summary of the plasma PK parameters for daunorubicin and daunorubicinol is presented in Supplementary Table S1. Based on the ANOVA analysis, the point estimate of the geometric mean ratio for daunorubicin AUC_tau_ was approximately 3% lower for patients with severe renal impairment (Cohort 3) and 6% lower for patients with moderate renal impairment (Cohort 2) compared with patients with normal renal function (Cohort 1). The point estimate of the geometric mean ratio for daunorubicin C_max_ was approximately 25% and 22% lower for patients with severe and moderate renal impairment (Cohorts 3 and 2, respectively) compared with patients with normal renal function (Cohort 1) (Supplementary Table S1). Based on the results of the primary ANOVA analyses, these differences were within the observed variability of AUC_tau_ and C_max_, suggesting that renal impairment did not have a clinically significant impact on the daunorubicin exposure (Table 2). The daunorubicinol exposure (AUC_tau_ and C_max_) was not substantially affected with increasing levels of renal impairment. For daunorubicin, the Ae was slightly lower with increasing renal impairment; however, both the Ae and CLR were low for daunorubicin across all three cohorts, indicating the minimal contribution of renal elimination (Table 3). The Ae% values for daunorubicin and daunorubicinol were <9% across all the cohorts, indicating a low contribution of renal elimination, and increasing renal impairment only contributed an approximate 5.6% reduction in daunorubicin and daunorubicinol Ae% (Table 3).

### 3.4. Safety and Tolerability

There were no significant deteriorations in renal function and the creatinine levels remained relatively stable during the study across cohorts: some patients exhibited shifts (either downward or upward) to Grade 1 and Grade 2 creatinine levels over time, but no participants exhibited shifts to ≥Grade 3 levels and no patients required dialysis during the study.

The most common (≥20%) TEAEs by cohort and overall are summarized in Table 4. All the patients reported ≥1 TEAE and the incidence of serious and severe TEAEs was similar across all the cohorts. Furthermore, all the patients reported ≥1 Grade 3–5 TEAE; however, no patients discontinued treatment due to TEAEs. The most frequently reported TEAEs were febrile neutropenia (57%), nausea (57%), and hyperglycemia (43%). Five (71%) patients with normal renal function (Cohort 1) had febrile neutropenia, compared with six (75%) patients with moderate renal impairment (Cohort 2) and one (17%) patient with severe renal impairment (Cohort 3) (Table 4). Serious TEAEs were reported by three (43%), three (38%), and two (33%) patients with normal renal function (Cohort 1), moderate, and severe renal impairment (Cohorts 2 and 3), respectively, whereas three patients (one from Cohort 1 and two from Cohort 2) experienced TEAEs that led to death, all occurring during the induction phase. The full dose of CPX-351 was tolerated in the first three patients with severe renal impairment (Cohort 3), and therefore, no dose reduction was deemed necessary for the remaining patients in this cohort, and patients received the planned full dose.

### 3.5. Efficacy

Overall, 52.4% of patients achieved a CR or a CRi based on the investigator-assessed unconfirmed post-induction responses (Table 5). A CR or CRi response was achieved by 57.2% of patients with normal renal function (Cohort 1); 50% of patients with moderate renal impairment (Cohort 2; two patients not assessed); and 50% of patients with severe renal impairment (Cohort 3; one patient not assessed).

## 4. Discussion

In this phase 1 study, the PK and safety profile of CPX-351 were assessed in patients with hematologic malignancies and normal or impaired renal function. Although there were broad eligibility criteria, most patients (86%) had a diagnosis of AML, two patients (10%) presented with refractory/relapsed AML, and three (14%) patients had a diagnosis of MDS. Since CPX-351 was tolerated in the first three patients in Cohort 3 with severe impairment, the full standard dose of CPX-351 was administered to all the patients, regardless of their renal impairment status.

Renal impairment did not impact the exposures (AUC and C_max_) of the active parent cytarabine, but slight increases in the Ara-U exposure with increasing levels of renal impairment were observed. Since these slight increases in exposure were identified for an inactive metabolite, these changes were not considered clinically meaningful. The PK characteristics of daunorubicin and daunorubicinol were similar across the renal function cohorts and were not substantially impacted by renal impairment. These results are supported by a previous population PK analysis comparing exposures of CPX-351 in patients with hematologic malignancies with mild and moderate renal impairment vs. normal renal function. Wang et al. reported a similar mean AUC_tau_ between patients with normal renal function and mild/moderate renal impairment for daunorubicin and cytarabine in an analysis of data collected from three clinical studies of patients with advanced hematologic malignancies, AML or acute lymphocytic leukemia, and newly diagnosed high-risk/secondary AML [18].

Although not designed as a formal statistical comparison, there were no discernible differences observed in the safety outcomes of CPX-351 across the renal function cohorts, and our findings were generally consistent with the known safety profile of CPX-351 [7,15,16,20].

Overall, the unconfirmed response rates observed in this study (52.4% CR + CRi) were consistent with response data previously reported for CPX-351. In the phase 2 trial of CPX-351 in 126 patients aged 60–75 years newly diagnosed with untreated AML, CPX-351 treatment demonstrated a higher CR + CRi rate vs. 7 + 3 chemotherapy (66.7% vs. 51.2; *p* = 0.07); CR was achieved by 48.8% of patients in both arms and the CRi rate was higher with CPX-351 than with 7 + 3 chemotherapy (17.9% vs. 2.4%) [7]. Subsequently, in the phase 3 trial, which enrolled 309 patients aged 60–75 years with newly diagnosed high-risk AML, the CR + CRi response rates were higher for CPX-351-treated patients vs. those treated with 7 + 3 chemotherapy (47.7% vs. 33.3%; odds ratio 1.77 [95% CI: 1.11–2.81]; *p* = 0.016) [20], whereas in the 5-year extension study, the CR + CRi rate for CPX-351-treated patients (*n* = 153) was 48.0% [21]. However, given the differences in patient population characteristics, it is difficult to draw direct comparisons between the responses observed in the present study and those observed in prior clinical trials.

The limitations of this study included the small sample sizes per cohort (which limits the statistical power for a number of analyses, meaning that statistical comparisons should be interpreted cautiously) and the fact that responses were not independently confirmed. Furthermore, this study did not evaluate the effect of hemodialysis on CPX-351 exposures. The initial trial exclusion criteria in oncology studies may disproportionally affect patients with the comorbidity of renal impairment [22].

## 5. Conclusions

Overall, in this study, we found that moderate or severe renal impairment did not have a clinically meaningful impact on the CPX-351 PK parameters or safety profile. The CPX-351 parent (total cytarabine and daunorubicin) exposures in patients with moderate and severe renal impairment were similar to those in patients with normal renal function. Based on the limited impact of renal impairment on CPX-351 exposure in patients with hematologic malignancies, and supported by exposure–response analyses of CPX-351 efficacy and safety in this study, adjustment of the CPX-351 dose in patients with mild, moderate, or severe renal impairment is not warranted. These data support the inclusion of patients with renal impairment in future randomized controlled oncology trials to better reflect expected drug toxicities and determine treatment efficacy and safety in specific patient populations.

## Figures and Tables

**Table 1:** Patient demographics and baseline characteristics (safety analysis set).

	Cohort 1: Normal Renal Function (*n* = 7)	Cohort 2: Moderate Renal Impairment (*n* = 8)	Cohort 3: Severe Renal Impairment (*n* = 6)
Median age at baseline, years (min, max)	67 (62, 76)	69 (60, 77)	74 (58, 85)
Male, *n* (%)	5 (71)	6 (75)	3 (50)
Diagnosis, *n* (%)			
AML	5 (71)	7 (88)	6 (100)
Newly diagnosed	5 (71)	6 (75)	5 (83)
De novo AML	2 (29)	2 (25)	4 (67)
Secondary AML	3 (43)	4 (50)	1 (17)
Refractory/relapsed	0	1 (13)	1 (17)
MDS	2 (29)	1 (13)	0
Race, *n* (%)			
Black or African American	0	2 (25)	1 (17)
White	7 (100)	6 (75)	5 (83)
Ethnicity, *n* (%)			
Not Hispanic or Latino	7 (100)	8 (100)	6 (100)
BSA (m^2^) ^a^			
Mean (SD)	2.27 (0.16)	1.98 (0.20)	1.83 (0.30)
ECOG Performance Status, *n* (%)			
0	2 (29)	0	0
1	2 (29)	7 (88)	3 (50)
2	3 (43)	1 (13)	3 (50)
CrCl (mL/min)			
Mean (SD)	125.89 (28.73)	47.49 (5.79)	23.20 (4.12)
eGFR (mL/min/1.73 m^2^)			
Mean (SD)	86.00 (18.61)	40.23 (8.51)	21.86 (2.21)

^a^ BSA (m^2^) data were not available for one patient in Cohort 1 (normal) and two patients in Cohort 2 (moderate). AML, acute myeloid leukemia; BSA, body surface area; CrCl, creatinine clearance; ECOG, Eastern Cooperative Oncology Group; eGFR, estimated glomerular filtration rate; Max, maximum; MDS, myelodysplastic syndrome; Min, minimum; SD, standard deviation.

**Table 2:** Statistical analysis of the impact of renal impairment on the pharmacokinetics of CPX-351 (PK evaluable analysis set).

		Ratio (%) of Test/Reference		
Analyte Comparison	PK Parameter	Estimate	90% CI	CV%
Cytarabine				
Cohort 2 vs. Cohort 1	AUC_tau_ (h × ng/mL)	91.56	(43.62–192.18)	98.9
	C_max_ (ng/mL)	79.60	(51.68–122.60)	51.1
Cohort 3 vs. Cohort 1	AUC_tau_ (h × ng/mL)	100.38	(45.24–222.74)	98.9
	C_max_ (ng/mL)	79.28	(49.83–126.13)	51.1
Daunorubicin				
Cohort 2 vs. Cohort 1	AUC_tau_ (h × ng/mL)	94.44	(52.24–170.73)	73.9
	C_max_ (ng/mL)	77.53	(52.36–114.78)	45.9
Cohort 3 vs. Cohort 1	AUC_tau_ (h × ng/mL)	97.04	(51.35–183.40)	73.9
	C_max_ (ng/mL)	74.54	(48.88–113.65)	45.9

An analysis of variance (ANOVA) was performed on the ln-transformed AUC_tau_, C_max_, CL_SS_, and CLR using PROC MIXED in SAS. Results are based on the linear fixed-effect model with a term for the cohort (renal function). AUC_tau_, area under the plasma concentration–time curve over the dosing interval; CI, confidence interval; C_max_, maximum observed concentration; CV, coefficient of variation; PK, pharmacokinetic; SAS, Statistical Analysis System.

**Table 3:** Urinary excretion parameters of the individual components of CPX-351 and their respective metabolites (PK evaluable analysis set).

Parameter ^a^	Cohort 1: Normal Renal Function (*n* = 7)	Cohort 2: Moderate Renal Impairment (*n* = 8)	Cohort 3: Severe Renal Impairment (*n* = 6)
Cytarabine			
Ae (µg)	2066 (1856 ± 2163) ^b^	1524 (1203 ± 889.6)	NC (NC)
CLR (mL/h)	1.387 (1.873 ± 2.645) ^c^	1.688 (2.526 ± 4.842)	NC (NC) ^c^
Ara-U			
Ae (µg)	121,900 (123,000 ± 17,750)	126,700 (132,200 ± 39,680)	117,100 (123,000 ± 41,010)
Cytarabine and Ara-U			
Ae_total	123,200 (124,500 ± 19,740)	127,200 (132,800 ± 40,080)	116,600 (122,500 ± 40,780)
Ae%	55.80 (56.05 ± 5.941)	64.98 (68.04 ± 21.46)	63.70 (67.32 ± 21.63)
Daunorubicin			
Ae (µg)	2481 (2567 ± 675.9)	1314 (1345 ± 309.1)	718.8 (899.6 ± 654.0)
CLR (mL/h)	4.810 (5.942 ± 3.719) ^d^	2.653 (4.410 ± 6.325)	1.381 (1.777 ± 1.365) ^d^
Daunorubicinol			
Ae (µg)	5944 (6015 ± 982.2)	3570 (3936 ± 1865)	1586 (1761 ± 877.3)
Daunorubicin and daunorubicinol			
Ae_total	8432 (8563 ± 1594) ^d^	4937 (5265 ± 2035)	2332 (2654 ± 1497) ^d^
Ae%	8.469 (8.577 ± 1.458) ^d^	5.733 (6.053 ± 2.027)	2.893 (3.242 ± 1.737) ^d^

^a^ All values are presented as the geometric mean (arithmetic mean ± standard deviation). ^b^ For cytarabine Ae: Cohort 1 (normal), *n* = 6. ^c^ For cytarabine CLR: Cohort 1 (normal), *n* = 6; Cohort 3 (severe), *n* = 5. ^d^ For daunorubicin CLR and for daunorubicin and daunorubicinol Ae_total and Ae%: Cohort 1 (normal), *n* = 6; Cohort 3 (severe), *n* = 5. Ae, cumulative amount of drug or metabolite eliminated in the urine over 48 h; Ara-U, arabinosyluracil; CLR, renal clearance; NC, not calculated; PK, pharmacokinetic.

**Table 4:** Summary of the most common (≥20%) TEAEs by cohort and overall (safety analysis set).

	Cohort 1: Normal Renal Function (*n* = 7)	Cohort 2: Moderate Renal Impairment (*n* = 8)	Cohort 3: Severe Renal Impairment (*n* = 6)	Overall (*N* = 21)
Number of TEAEs, *n*	141	170	166	477
Number of patients with ≥1 TEAE, *n* (%)	7 (100)	8 (100)	6 (100)	21 (100)
Febrile neutropenia	5 (71.4)	6 (75.0)	1 (16.7)	12 (57.1)
Nausea	4 (57.1)	5 (62.5)	3 (50.0)	12 (57.1)
Hyperglycemia	3 (42.9)	2 (25.0)	4 (66.7)	9 (42.9)
Anemia	4 (57.1)	2 (25.0)	2 (33.3)	8 (38.1)
Fatigue	2 (28.6)	4 (50.0)	2 (33.3)	8 (38.1)
Leukopenia	3 (42.9)	2 (25.0)	3 (50.0)	8 (38.1)
Headache	4 (57.1)	2 (25.0)	1 (16.7)	7 (33.3)
Hypertension	2 (28.6)	2 (25.0)	3 (50.0)	7 (33.3)
Hypokalemia	3 (42.9)	1 (12.5)	3 (50.0)	7 (33.3)
Hyponatremia	3 (42.9)	2 (25.0)	2 (33.3)	7 (33.3)
Lymphopenia	2 (28.6)	2 (25.0)	3 (50.0)	7 (33.3)
Edema peripheral	3 (42.9)	2 (25.0)	2 (33.3)	7 (33.3)
Blood bilirubin increased	3 (42.9)	2 (25.0)	1 (16.7)	6 (28.6)
Constipation	2 (28.6)	2 (25.0)	2 (33.3)	6 (28.6)
Hypocalcemia	1 (14.3)	2 (25.0)	3 (50.0)	6 (28.6)
Neutropenia	1 (14.3)	2 (25.0)	3 (50.0)	6 (28.6)
Thrombocytopenia	2 (28.6)	2 (25.0)	2 (33.3)	6 (28.6)
Aspartate aminotransferase increased	2 (28.6)	2 (25.0)	1 (16.7)	5 (23.8)
Contusion	2 (28.6)	1 (12.5)	2 (33.3)	5 (23.8)
Decreased appetite	2 (28.6)	1 (12.5)	2 (33.3)	5 (23.8)

TEAE, treatment-emergent adverse event. Adverse events were coded using the Medical Dictionary for Regulatory Activities Version 21.0. Percentages were calculated based on the number of participants in each treatment course and cohort. If a participant experienced more than one event in a given preferred term (PT), that participant was counted once for that PT.

**Table 5:** Investigator-assessed post-induction unconfirmed responses by cohort (safety analysis set).

Response, *n* (%)	Cohort 1: Normal Renal Function (*n* = 7)	Cohort 2: Moderate Renal Impairment (*n* = 8)	Cohort 3: Severe Renal Impairment (*n* = 6)	Overall (*N* = 21)
CR	2 (28.6)	1 (12.5)	3 (50.0)	6 (28.6)
CRi	2 (28.6)	3 (37.5)	0	5 (23.8)
PD	3 (42.9)	2 (25.0)	2 (33.3)	7 (33.3)
Relapsed	0	0	0	0
Not assessed	0	2 (25.0)	1 (16.7)	3 (14.3)

CR, complete remission; CRi, complete remission with incomplete platelet or neutrophil recovery; PD, persistent disease.

## Data Availability

All relevant data are provided within the manuscript and supporting files. The datasets generated during and/or analyzed during the current study are available from the corresponding author on reasonable request.

## Supplementary Materials

The following supporting information can be downloaded at https://www.mdpi.com/article/10.3390/cancers16050915/s1, Figure S1: Patient disposition; Table S1: Plasma pharmacokinetic parameters of the individual components of CPX-351 and their respective metabolites on day 5 (PK evaluable analysis set).
