# Supplementary material for: CPX-351 Pharmacokinetics and Safety in Adults with Hematologic Malignancies and Renal Function Impairment: Phase 1 Trial
Source: Cancers (Basel). 2024 Feb 24;16(5):915. doi: 10.3390/cancers16050915 (PMC10931427; doi:10.3390/cancers16050915)
Supplement: Supplementary file 1 [file cancers-16-00915-s001.zip › cancers-2763111-supplementary.pdf]

cancers

Supplementary Materials

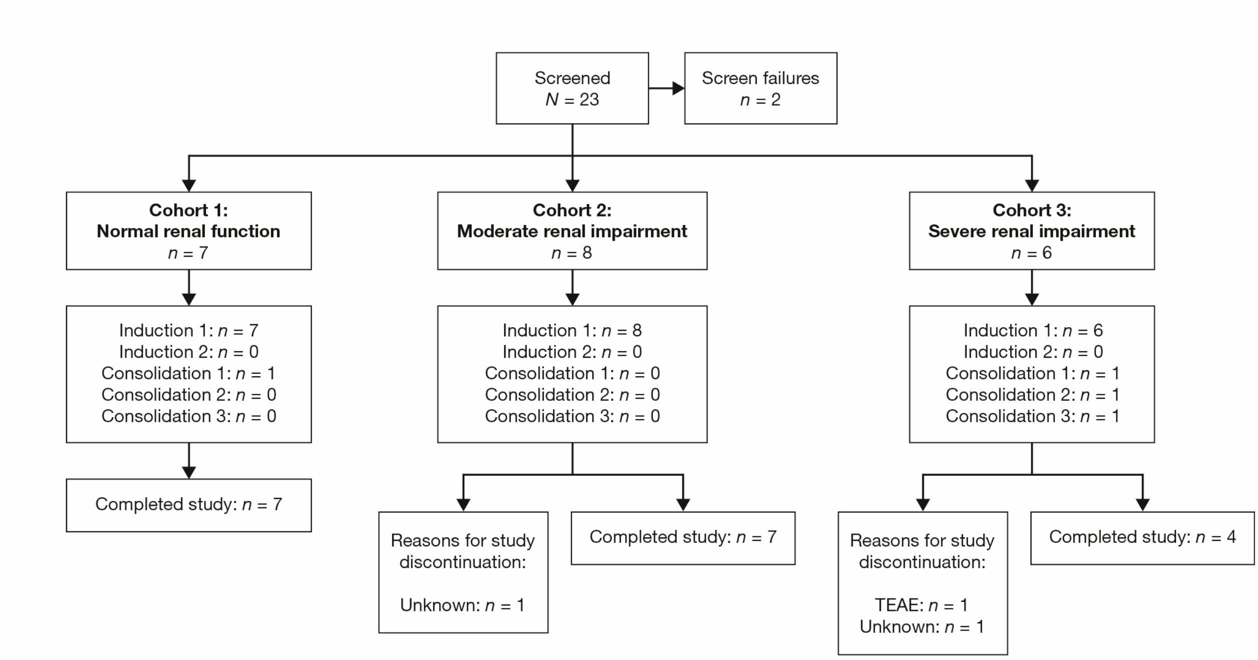

Figure S1. Patient disposition.

Table S1. Plasma pharmacokinetic parameters of the individual components of CPX-351 and their respective metabolites on day 5 (PK evaluable analysis set).

Parameter[a]	Cohort 1: Normal Renal Function (n = 7)	Cohort 2: Moderate Renal Impairment (n = 8)	Cohort 3: Severe Renal Impairment (n = 6)
Cytarabine			
AUC_{tau} (h×ng/mL)	1,325,000 (1,549,000 ± 920,200)	1,213,000 (1,704,000 ± 1,004,000)	1,330,000 (1,342,000 ± 203,100)
C_{max} (ng/mL)	65,740 (74,260 ± 41,250)	52,330 (58,430 ± 21,890)	52,120 (52,380 ± 5,973)
T_{max} (h)	2.00 (0.60, 5.80)	2.00 (1.50, 24.40)	2.00 (2.00, 4.02)
$T_{1/2}$ (h)	25.94 (31.71 ± 17.97)	24.47 (28.42 ± 15.35)	29.46 (29.92 ± 5.550)
CL_{ss} (mL/h)	168.1 (204.1 ± 138.9)[b]	161.4 (380.9 ± 744.2)	135.5 (139.1 ± 31.36)
Ara-U			
$AUC_{0\text{-}48}$ (h×ng/mL)	37,660 (38,760 ± 10,100)	57,670 (59,400 ± 15,390)	76,700 (82,030 ± 31,220)
C_{max} (ng/mL)	1,113 (1,151 ± 339.9)	1,738 (1,888 ± 899.3)	1,836 (1,920 ± 599.5)
T_{max} (h)	8.00 (7.62, 8.07)	6.76 (2.97, 24.40)	7.89 (6.00, 8.00)
$T_{1/2}$ (h)	52.75 (56.73 ± 20.53)	45.19 (50.13 ± 22.92)	44.31 (46.77 ± 19.28)
Ratio_M/P	0.02830 (0.03111 ± 0.01526)	0.04735 (0.1196 ± 0.2455)	0.05739 (0.06198 ± 0.02751)
Daunorubicin			
AUC_{tau} (h×ng/mL)	524,100 (604,900 ± 352,100)	495,000 (617,100 ± 311,000)	508,600 (518,000 ± 110,300)
C_{max} (ng/mL)	29,900 (34,670 ± 23,520)	23,180 (25,000 ± 8,513)	22,290 (22,500 ± 3,486)
T_{max} (h)	2.00 (0.60, 4.13)	2.00 (1.52, 2.32)	2.00 (1.50, 2.00)
$T_{1/2}$ (h)	17.40 (21.38 ± 13.09)	19.44 (21.85 ± 10.33)	21.41 (22.08 ± 5.794)
CL_{ss} (mL/h)	189.3 (220.6 ± 130.3)	173.8 (267.0 ± 366.9)	156.1 (162.1 ± 44.86)
Daunorubicinol			
$AUC_{0\text{-}48}$ (h×ng/mL)	5,063 (5,844 ± 3,241)	6,634 (7,844 ± 4,600)	4,683 (4,750 ± 861.2)
C_{max} (ng/mL)	121.0 (136.3 ± 71.16)	197.7 (248.2 ± 167.4)	106.5 (107.9 ± 18.61)
T_{max} (h)	4.13 (0.60, 48.25)	16.90 (1.50, 48.05)	24.05 (0.00, 48.08)
$T_{1/2}$ (h)	44.26 (46.96 ± 19.37)	36.90 (38.40 ± 11.91)[b]	43.95 (44.92 ± 11.0)[b]
Ratio_M/P	0.009624 (0.009727 ± 0.001572)	0.01337 (0.01531 ± 0.008372)	0.009178 (0.009287 ± 0.001608)

[a]All values are presented as geometric mean (arithmetic mean ± SD) except for T_{max}, which is presented as median (min, max)
[b]For CL_{ss}: Cohort 1 (normal), n = 5. Ara-U, arabinosyluracil; $AUC_{0\text{-}48}$, area under the plasma concentration-time curve from time 0 to the time of the next dosing during a 48-hour interval; AUC_{tau}, area under the plasma concentration-time curve over the dosing interval; CL_{ss}, clearance at steady-state; C_{max}, maximum observed concentration; Max, maximum; Min, minimum; PK, pharmacokinetic; Ratio_M/P, $AUC_{0\text{-}48}$ for the respective metabolite/AUC_{tau} for the respective parent drug; $T_{1/2}$, terminal half-life; T_{max}, time of C_{max}.
